# Simulation-based assessment of robotic cardiac surgery skills: An international multicenter, cross-specialty trial

**DOI:** 10.1016/j.xjon.2023.10.029

**Published:** 2023-11-02

**Authors:** Gennady V. Atroshchenko, Emiliano Navarra, Matthew Valdis, Elena Sandoval, Nasseh Hashemi, Stepan Cerny, Daniel Pereda, Meindert Palmen, Flemming Bjerrum, Niels Henrik Bruun, Martin G. Tolsgaard

**Affiliations:** a Department of Cardiothoracic Surgery, Aalborg University Hospital, Aalborg, Denmark; b ROCnord Robotic Centre Aalborg, Aalborg University Hospital, Aalborg, Denmark; c Department of Clinical Medicine, Aalborg University, Aalborg, Denmark; d Department of Cardiac Surgery, Ospedale Sant'Andrea, “Sapienza” University of Rome, Rome, Italy; e Division of Cardiac Surgery, Department of Surgery, Western University, London Health Sciences Center, London, Ontario, Canada; f Department of Cardiovascular Surgery, Hospital Clínic, Barcelona, Spain; g Nordsim, Aalborg University Hospital, Aalborg, Denmark; h Department of Cardiac Surgery, Na Homolce Hospital, Prague, Czech Republic; i Centro de Investigación Biomédica en Red de Enfermedades Cardiovasculares (CIBERCV), Madrid, Spain; j Department of Cardiothoracic Surgery, Leiden University Medical Center, Leiden, The Netherlands; k Department of Cardiothoracic Surgery, Amsterdam University Medical Center, Amsterdam, The Netherlands; l Department of Gastrointestinal and Hepatic Diseases, Copenhagen University Hospital–Herlev and Gentofte, Herlev, Denmark; m Copenhagen Academy for Medical Education and Simulation (CAMES), Rigshospitalet, Denmark; n Unit of Clinical Biostatistics, Aalborg University Hospital, Aalborg, Denmark; o Department of Obstetrics, Copenhagen University Hospital Rigshospitalet, Denmark; p Department of Medicine, University of Copenhagen, Denmark

**Keywords:** robotic cardiac surgery, assessment, validity, wet lab, simulation

## Abstract

**Objective:**

This study aimed to investigate the validity of simulation-based assessment of robotic-assisted cardiac surgery skills using a wet lab model, focusing on the use of a time-based score (TBS) and modified Global Evaluative Assessment of Robotic Skills (mGEARS) score.

**Methods:**

We tested 3 wet lab tasks (atrial closure, mitral annular stitches, and internal thoracic artery [ITA] dissection) with both experienced robotic cardiac surgeons and novices from multiple European centers. The tasks were assessed using 2 tools: TBS and mGEARS score. Reliability, internal consistency, and the ability to discriminate between different levels of competence were evaluated.

**Results:**

The results demonstrated a high internal consistency for all 3 tasks using mGEARS assessment tool. The mGEARS score and TBS could reliably discriminate between different levels of competence for the atrial closure and mitral stitches tasks but not for the ITA harvesting task. A generalizability study also revealed that it was feasible to assess competency of the atrial closure and mitral stitches tasks using mGEARS but not the ITA dissection task. Pass/fail scores were established for each task using both TBS and mGEARS assessment tools.

**Conclusions:**

The study provides sufficient evidence for using TBS and mGEARS scores in evaluating robotic-assisted cardiac surgery skills in wet lab settings for intracardiac tasks. Combining both assessment tools enhances the evaluation of proficiency in robotic cardiac surgery, paving the way for standardized, evidence-based preclinical training and credentialing.

**Clinical trial registry number:**

NCT05043064.

## Graphical abstract

**Figure:**
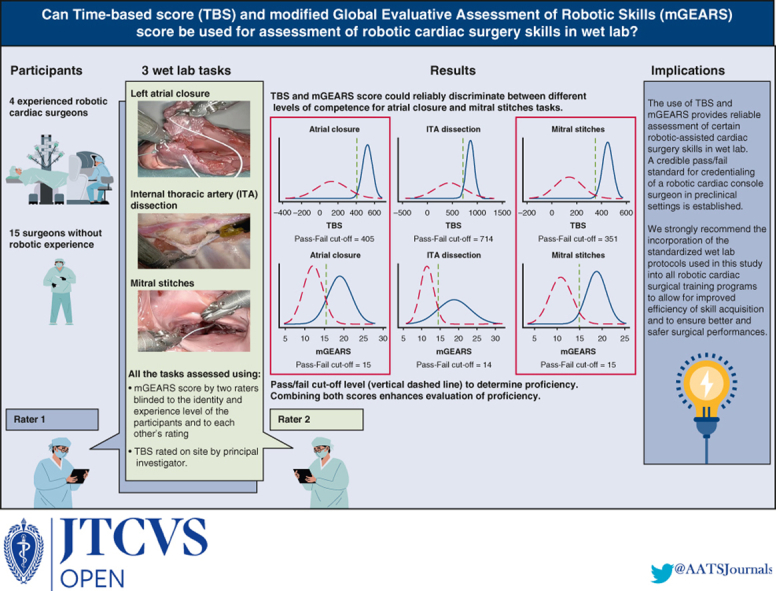

**Figure:**
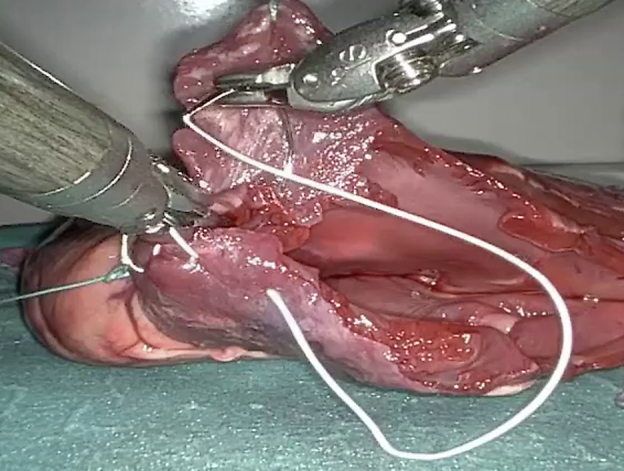
Robotic-assisted closure of the porcine atrium.

**Central Message**

The use of time-based score and a modified GEARS score provides reliable assessments of certain robotic-assisted cardiac surgery skills with sufficient validity evidence for wet lab settings.

**Perspective**

As robotic-assisted cardiac surgery requires a specific skill set, there is a rising demand for standardized training pathways. The study provides validity evidence for using a time-based score and modified GEARS score in evaluating certain robotic-assisted cardiac surgery skills in wet lab settings. A reliable pass/fail standard for credentialing of a robotic cardiac console surgeon was established.

Robotic-assisted surgery has entered the mainstream of cardiac surgery, with large numbers of cases now performed at specialized centers worldwide.^1^, ^2^, ^3^ Despite the demonstrated benefits and the growing number of robotic-assisted procedures, the adoption of this technology has been slow, partly due to a lack of structured training programs.^4^^,^^5^ As robotic-assisted surgery requires a specific skill set, there is a rising demand for standardized international training pathways to prepare the next generation of robotic surgeons.^6^ Yet, robotic-assisted cardiac surgery skill training is minimal or absent in residency curricula, and there are no credentialing requirements for board certification.^5^^,^^7^

Simulation-based training has been increasingly used to train surgeons in preclinical settings^8^ and to assess competency.^9^, ^10^, ^11^ Simulation-based training enables skill development outside of the operating room, allowing for feedback and correction of errors in a controlled and safe environment without any risk to patients.^12^ In the simulated setting, trainees can practice until they reach a standard level of competency before beginning supervised clinical training. However, in order to determine when trainees have achieved this level of competency, assessments with an established validity evidence are necessary.^13^ The objective of this study was to investigate the validity of simulation-based assessment of robotic-assisted cardiac surgery skills in a wet lab setting.

## Methods

**Figure 1:**
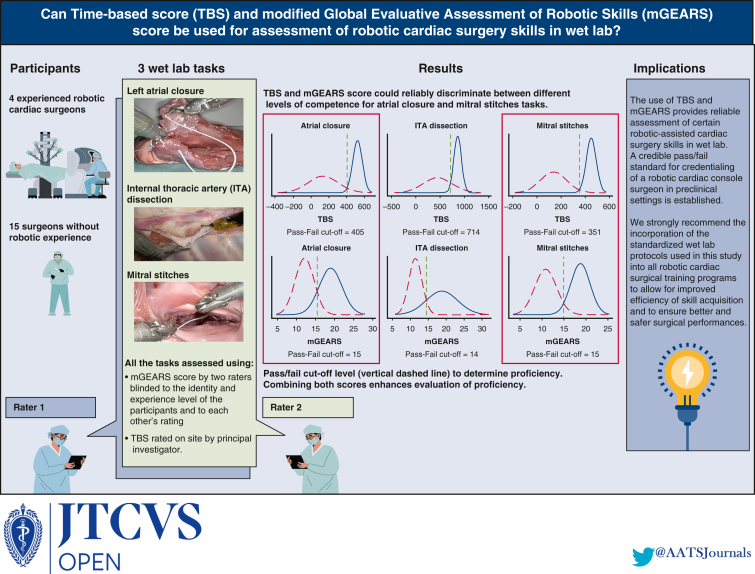
Simulation-based assessment of robotic cardiac surgery skills.

This was a prospective study gathering data between September 27 and December 2, 2021, at the Robotic Centre ROCnord, Biomedical Research Laboratory, Aalborg University (Figure 1). A contemporary framework of validity^14^ was used to describe the evidence for the assessment of robotic-assisted cardiac surgery skills in a wet lab model. Five sources of validity evidence were gathered: content evidence, response process, internal structure, relation to other variables, and consequences of testing.

The Regional Scientific Ethical Committee of the North Denmark Region assessed that approval was not required for this study (reference number 2020-000992-55). The study was registered at ClinicalTrials.gov (NCT05043064). All participants gave verbal and signed informed consent, including agreeing to video recordings.

### Study Context

The study was conducted in an animal wet lab using the da Vinci Xi Surgical System (Intuitive Surgical). All procedures were video-recorded and coded for blinded assessment. A time-based score (TBS) and a modified Global Evaluative Assessment of Robotic Skills (mGEARS) score (Table 1) were employed to evaluate participants' performance. In mGEARS, the performances were not rated on the autonomy domain—a part of the full GEARS^15^ score—as autonomy could not be evaluated from the recordings. Two experienced robotic cardiac surgeons from Canada and Spain rated all videos using mGEARS independently, whereas TBS was rated on-site by the principal author. The raters were blinded to the identity and experience level of the participants, and to each other's ratings.

**Table 1:** Modified global evaluative assessment of robotic skills (mGEARS)^15^

**Depth perception**				
1	2	3	4	5
Constantly overshoots target, wide swings, slow to correct		Some overshooting or missing target, but quick to correct		Accurately directs instruments in the correct plane to target
**Bimanual dexterity**				
1	2	3	4	5
Uses only one hand, ignores nondominant hand, poor and coordination		Uses both hands but does no optimize interaction between hands		Expertly uses both hands in a complementary way to provide best exposure
**Efficiency**				
1	2	3	4	5
Inefficient efforts; many uncertain movements, constantly changing focus or persisting without progress		Slow, but planned movement are reasonably organized		Confident, efficient, and safe conduct, maintains focus on task, fluid progression
**Force sensitivity**				
1	2	3	4	5
Rough moves, tears tissue, injures nearby structures, poor control, frequent suture breakage		Handles tissues reasonably well, minor trauma to adjacent tissue, rare suture breakage		Applies appropriate tension, negligible injury to adjacent structures, no suture breakage
**Robotic control**				
1	2	3	4	5
Consistently does not optimize view, hand position, or repeated collisions even with guidance		View is sometime not optimal. Occasionally needs to relocate arms. Occasional collisions and obstruction of assistant.		Controls camera and hand position optimally and independently. Minimal collisions or obstruction of assistant.

### Outcomes

The primary outcome was TBS and the secondary outcome was the mGEARS score for each task. The time-based score equation (adapted from Valdis and colleagues^16^) was calculated in seconds as follows: TBS = max time − completion time − errors. Max time was the maximum total time that a participant spent to complete the task during the first attempt. Completion time was the time used by each study participant to finish the task. Errors were divided into minor and major ones. A minor error resulted in adding a 10-second penalty time to the TBS equation. A major error led to a score of zero, regardless of time to completion.

Minor errors included moving robotic arms out of view, robotic arm collision, dropping the needle. Major errors included avulsion of internal thoracic artery (ITA) or suturing model, tearing/fraying the suture, and gross tissue damage (specifically with the cautery).

### Setting up Cases

The da Vinci Xi Surgical System with Xi/X 8-mm endoscope plus, 30° was used. Prepared porcine anterior chest wall with the intact sternum and attached costae in a reversed “V” shape was used for robotic-assisted harvesting of ITA. The fascia and covering intercostal muscles were removed to expose the ITA and the internal thoracic vein (anatomically there is only one corresponding vein in porcine and both artery and vein are covered a thick layer of intercostal muscles in contrast to human). An isolated porcine heart from 35- to 60-kg pigs was used for robotic-assisted mitral surgery. An 8-cm left atriotomy was made to provide access to the mitral valve. Two sutures at each corner of the atriotomy were placed to suspend the atrial opening and a single loose suture mimicking robotic dynamic atrial retractor was additionally used to elevate the upper part of the atriotomy. The prepared chest plate and the heart were installed in the da Vinci Simulator Box, which had multiple orifices for the robotic arm placement.

The experienced robotic cardiac surgeons participating in the study were asked to select 3-part procedures from real robotic cardiac operations that they believed were most suitable for wet lab training for a console surgeon. The following tasks were chosen (Video 1):

1. Robotic-assisted closure of the porcine atrium. Before task assessment, two 15-cm-long Gore-Tex CV-3 sutures (W.L. Gore & Associates) were placed at each corner of an 8-cm-long left atriotomy of the porcine atrium. The participant then was required to suture the left atrium from both ends towards the middle of the atriotomy using robotic DeBakey forceps and a needle driver leaving both sutures untied. Decision to skip a robotic-assisted knots tying was made deliberately, as at some institutions it is done nonrobotically by a bedside assistant and we aimed to focus solely on the console surgeon in order to make the task reproducible across the different centers.
2. Robotic-assisted placement of 5 sutures in the mitral annulus of the porcine heart. A single 8-cm-long 2-0 PremiCron suture (B. Braun Surgical) was passed to the participant by an assistant and needed to be placed through the posteromedial, anterolateral trigone, P1, P2, and P3 segment of the porcine mitral valve using the aforementioned robotic instruments. After passing each segment, the distal portion of the suture was cut by the participant allowing uninterrupted placement of the same suture without involvement of the assistant and thus entirely concentrating on the console surgeon performance.
3. Robotic-assisted harvesting of ITA. A 10-cm segment of ITA marked with a suture at both ends needed to be dissected from the porcine chest wall by the participant using robotic DeBakey forceps and monopolar cautery hook.

### Procedure

The experienced robotic cardiac surgeons familiarized themselves with the wet lab simulator and then performed each task 5 times. The robotic novices received a brief introduction to the da Vinci Surgical System and were shown videos of the procedures performed by experienced surgeons, highlighting basic operative techniques and relevant anatomy. They were then asked to do a warm-up round without recording to familiarize themselves with the wet lab simulator under the supervision of an experienced robotic cardiac surgeon. Next, the novices completed all 3 exercises again (the first attempt) with the guidance of the same robotic surgeon, and this was used to assess their performance. The second attempt for all novices was scheduled at a three-week interval. During the study period, participants were not allowed to practice on any robotic simulators or participate in robotic-assisted surgery.

### Statistics

To determine reliability of the assessment, internal consistency and test/retest reliability were assessed. Test/retest reliability was evaluated for the first 2 attempts by comparing the TBS and mGEARS scores for all participants. The internal consistency of the mGEARS metrics was calculated for the total number of attempts for each task. Generalizability (G) theory^14^ was used to estimate the overall reliability of the mGEARS assessment tool based on the first 2 attempts. A decision (D) study^14^ for each task was conducted to generate various generalizability (G) coefficients and to explore the number of raters and repetitions needed to reach an acceptable G-coefficient.

The TBS and mGEARS scores between the experienced robotic surgeons and the robotic novices were compared for the first attempt. To investigate the consequence of testing, a pass/fail level was determined using the “contrasting groups” standard-setting method.^17^ Both the observed and theoretical false positives/negatives were calculated using the cumulative distribution function to account for a small number of participants.^18^

Statistical analysis was performed using STATA/MP 17.0 (StataCorp). Descriptive statistics were calculated and expressed as medians and interquartile intervals and compared by the Kruskal–Wallis test. Linear regression with robust variance estimation were used exploring relationships between variables. Reliability was reported as intraclass correlation coefficient (ICC). Cronbach alpha was used to report internal consistency reliability. Generalizability study model was defined as a crossed model (*p* × *a* × *r*) with a study participant (*p*) as the object of measurement and 2 random facets: attempts (*a*) and raters (*r*). A G-coefficient of 0.8 was considered as acceptable.

## Results

Surgeons and surgical trainees from 5 different specialties and different European sites were recruited for the study by e-mail invitation sent to their surgical departments. The experienced robotic surgeons (n = 4) were board-certified cardiac surgeons who had robotic volume ranging from 55 to 350 robotic-assisted cardiac operations and were recruited from the Czech Republic, Belgium, the Netherlands, and Spain. Robotic novices (n = 15) with less than 5 hours of experience on any robotic surgical system were recruited from departments of cardiothoracic surgery (n = 7), abdominal surgery (n = 4), urology (n = 1), gynecology (n = 2), and otolaryngology (n = 1) in Denmark. Table 2 shows descriptive data on 19 study participants.

**Table 2:** Baseline characteristics of participants (n = 19)

Characteristic	Novices	Experienced surgeons	Total	*P* value
N	15	4	19	
Age, y, median (IQR)	47 (37; 56)	44 (42; 53)	45 (42; 55)	.92
Sex (female), n	5	0	5	.18
Specialist (yes), n∗	12	4	16	.33
Work experience, median (IQR)	5 (2; 17)	13 (11; 26)	10 (3; 17)	.12

*IQR*, Interquartile range.

∗ Board-certified specialist.

### Content Evidence

The decision to use both the TBS and mGEARS assessment tool to measure robotic cardiac surgical skills was based on reported data for using these 2 scores for similar purposes in wet lab settings.^16^

### Response Process

Before performing the tasks, all participants were introduced to mGEARS and TBS scores including the penalties.

### Internal Structure

**Figure 2:**
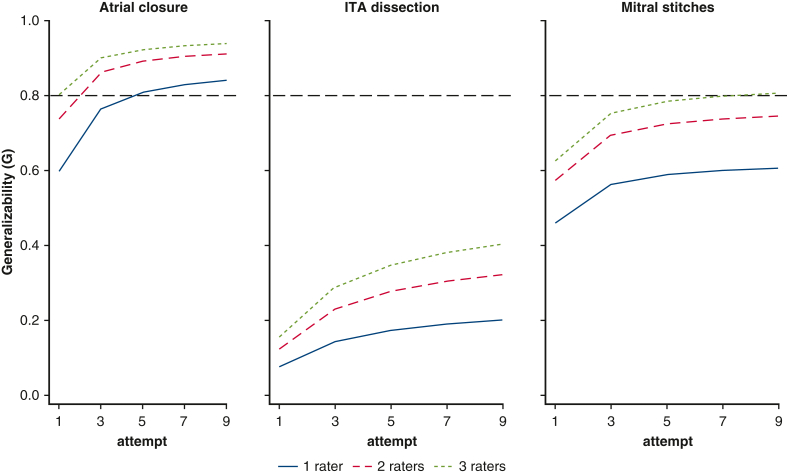
Number of attempts evaluated by raters in order to reach a G-coefficient of 0.8. *ITA*, Internal thoracic artery.

The test/retest reliability had an ICC of 0.7 for atrial closure, 0.45 for mitral stitches, and 0.37 for ITA dissection on TBS and an ICC of 0.77, 0.65, and 0.23 on mean mGEARS score for the same tasks, respectively. High internal consistency of 5 mGEARS items was found for all 3 tasks, with a Cronbach alpha of 0.91 for the atrial closure, 0.92 for the mitral stitches, and 0.92 for the ITA harvesting. A generalizability study (Table 3) revealed that the largest percentage of variance (47.2%) for the atrial closure task accounted for the participants, followed by 26.7% for the same trait for the mitral stitches task. The lowest number (4.2%) in the variation among the participants was found for the ITA task. Relatively high systemic variation among the raters indicated that the raters had different levels of leniency/stringency for all 3 exercises: 21% for atrial closure, 33.7% for mitral stitches, and 33% for ITA dissection. The results of the D-studies (Figure 2) demonstrated a G-coefficient of 0.82 with 2 raters and 2 attempts for the atrial closure task, and 3 raters and 6 attempts needed to reach a G-coefficient of 0.79 for the mitral stitches exercise. It would require 20 attempts and 25 raters to reach the former coefficient for the ITA dissection.

**Table 3:** The contribution of sources of variance to mGEARS assessment tool

Source of variance	Description	Atrial closure VC (%)	Mitral stitches VC (%)	ITA dissection VC (%)
Participant	“True score” variance for the participant	12.2 (47.2)	9.5 (26.7)	1.4 (4.2)
Attempt	Systematic variation among attempts	0 (0)	1.5 (4.4)	4 (12.2)
Raters	Systematic variation among raters	5.4 (21)	12 (33.7)	10.8 (32.9)
Interaction between participants and attempts	Inconsistencies in participants' performance between attempts	0.4 (1.6)	2.9 (8.3)	2.9 (8.9)
Interaction between participants and raters	Inconsistencies in raters' assessment of a participants' skill	1.5 (6.1)	5.5 (15.6)	4.1 (12.5)
Interaction between raters and attempts	Inconsistencies in attempt difficulty by rater	0 (0)	1.3 (3.7)	0 (0)
Residual error	All remaining variability	6.2 (24.1)	2.7 (7.6)	9.7 (29.3)

*VC*, Variance component; %, percent variance; *ITA*, internal thoracic artery.

### Relations to Other Variables

On the first attempt significant differences in both TBS and the average mGEARS score were found between the experienced robotic surgeons and the robotic novices for the mitral stitches and atrial closure tasks, but not for ITA harvesting (Table 4).

**Table 4:** Comparing novices' and experienced surgeons' performance at baseline

Characteristic	Novices mean ± SD	Experienced surgeons mean ± SD	Effect mean difference (95% CI)	*P* value
Atrial closure				
N	15	4		
TBS, s	135 ± 170	553 ± 47	−417 (−522 to −312)	<.001
mGEARS	12.1 ± 2.5	19.8 ± 5	−7.6 (−12.6 to −2.6)	.01
Mitral stitches				
N	15	4		
TBS, s	139 ± 108	425 ± 11	−286 (−347 to −225)	<.001
mGEARS	10.8 ± 2.5	19.6 ± 3.5	−8.8 (−12.5 to −5.1)	<.001
ITA harvesting				
N	15	3		
TBS, s	447 ± 300	638 ± 240	−192 (−496 to 113)	.2
mGEARS	11.4 ± 1.8	16.3 ± 5.5	−4.9 (−11 to 1)	.1

*SD*, Standard deviation; *CI*, confidence interval; *TBS*, time-based score; *mGEARS*, modified Global Evaluative Assessment of Robotic Skills; *ITA*, internal thoracic artery.

### Consequences

The maximum total time during the first attempt was 1222 seconds for the ITA task, 566 seconds for mitral sutures placement, and 769 seconds for the closure of the left atrium. These maximum times were used for subsequent calculation of TBS for each task as followed:

ITA dissection = 1222 − completion time − errors

Mitral stitches = 566 − completion time − errors

Atrial closure = 769 − completion time − errors.

**Figure 3:**
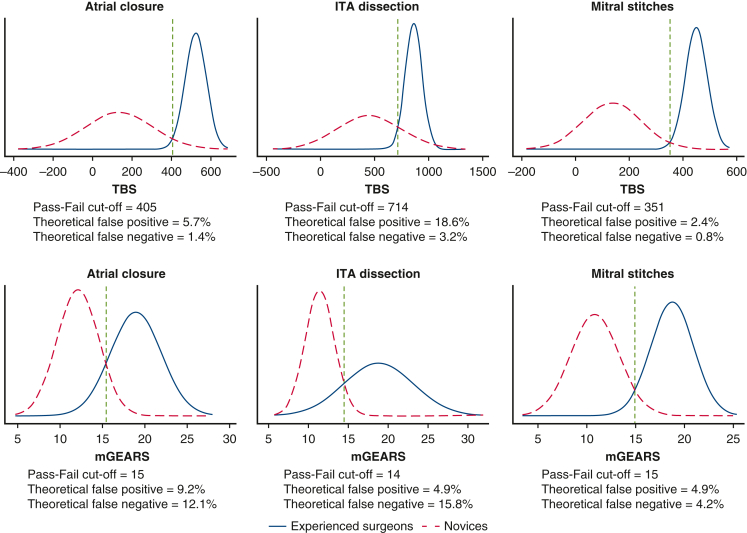
Determining pass/fail level using the “contrasting groups” method for time-based score (TBS) and modified global evaluative assessment of robotic skills score (*mGEARS*) in three wet lab tasks. *ITA*, Internal thoracic artery.

**Figure 4:**
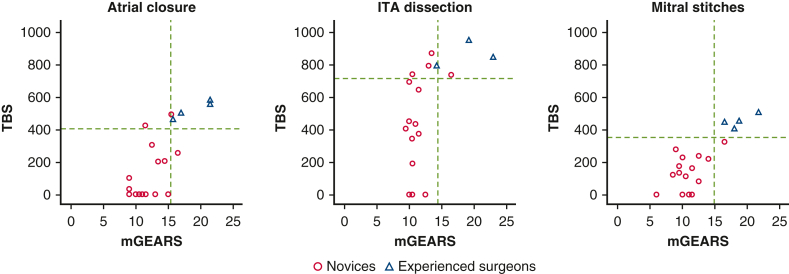
Pass/fail levels for time-based score (*TBS*) and modified global evaluative assessment of robotic skills score (*mGEARS*) combined against each other. *ITA*, Internal thoracic artery.

A pass/fail level for each task for both scores was established, and the theoretical numbers of passing novices (false positives) and failing experts (false negatives) were calculated to account for a small sample size (Figure 3). Combining the cut-off points for TBS and mGEARS assessment tools against each other (Figure 4) resulted in the following observed numbers: one passing novice and no failing experts for the atrial closure task, zero passing novices or failing experts for the mitral stitches exercise, and one passing novice and failing expert for the ITA dissection, based on blinded assessment.

## Discussion

In this study, we investigated the validity of a simulation-based assessment of robotic-assisted cardiac surgery skills using a wet lab model across surgeons from multiple different European centers. We tested 3 wet lab tasks using 2 assessment tools (TBS and mGEARS score) and demonstrated that 2 of 3 tasks could reliably discriminate between different levels of competence and can be used for competency assessment. We established a reliable pass/fail standard for each task, which can be used for credentialing of a robotic cardiac console surgeon in preclinical settings. We also investigated the validity of the modified GEARS score in wet lab settings, which supports its use in assessing overall robotic cardiac surgical skills. We recommend using both assessment tools to enhance evaluation of different aspects of competency in robotic cardiac surgical skills. To our knowledge, this is the first study to explore validity evidence for simulation-based assessment of robotic cardiac skills in a wet lab.

Currently, there are 2 pathways for robotic cardiac surgery wet lab training: robotic-assisted coronary revascularization and robotic-assisted intracardiac surgical training. Commonly used wet lab models include porcine,^16^ ovine,^19^ and cadavers.^20^ To our knowledge, there are no data comparing these models, and the choice of the model for robotic wet lab training depends on institutional routine, available resources, and legislation. Recent recommendations^21^ for robotic cardiac surgery training highlight wet lab laboratory training as an essential component. However, task selection can include various exercises for intracardial procedures such as sewing mitral bands or rings, neochordae implantation, valve resection, or atrial closure depending on the supervisor's choice. As surgical education is shifting toward competency-based education, there is a need for standard measurable settings for educational programs.^22^ In our study, we chose a porcine model for robotic cardiac surgery wet lab training as this model was previously investigated and is relatively inexpensive and easily available in most instances. The 3 investigated tasks covered both robotic cardiac training pathways—the robotic-assisted coronary revascularization (ITA harvesting) and intracardiac surgery (mitral stitches and atrial closure).

The Global Evaluative Assessment of Robotic Skills (GEARS) score is considered a reliable assessment tool for assessing general robotic skills.^15^ The assessment instrument is not specific to any particular robotic surgical procedure, and validity evidence for its use in robotic cardiac surgery has not previously been evaluated. By examining the internal structure of the exercises, we found a high internal consistency of the 5 investigated mGEARS domains for all 3 tasks, which is in agreement with the original GEARS work^15^ and indicates that even though the domains measure different characteristics (depth perception, bimanual dexterity, efficiency, force sensitivity, and robotic control), they are related to a single construct—robotic cardiac technical skills in the wet lab settings. We found that the mGEARS assessment tool can distinguish between novices and experienced robotic surgeons at the baseline in the atrial closure and mitral stitches tasks. However, in contrast to the existing literature,^16^ the difference among the groups was not statistically significant for the ITA exercise in our study. The score claims to have low variability among evaluators,^15^ but we were not able to confirm these findings in the generalizability study demonstrating high systematic variation among the raters ranging from 21% to 33% depending on the task and revealing different stringency between the raters. One plausible explanation for this finding could be that the raters were not trained in the GEARS evaluation before the study started, which has implications for how GEARS may be used by untrained raters in the future. In contrast, there were nearly no inconsistencies in the raters' assessment of the attempt difficulty. The D study showed that competency in robotic cardiac surgical skills using the mGEARS assessment tool can be assessed in a valid way by 2 raters and 2 attempts for the atrial closure and by 3 raters and 6 attempts for the mitral stitches. Based on our data, it does not seem feasible to assess competency in the ITA dissection validly using the mGEARS score. Overall, our findings support the use of the mGEARS score as a measure of competence for the assessment of robotic-assisted intracardiac surgical training.

Another important consideration is that mGEARS does not take time into account, which is crucial for cardiac surgical procedures, as the duration of aorta crossclamping and extracorporeal circulation influence the outcomes of the procedures.^23^ It necessitates adding time for the assessment of robotic cardiac surgical skills. In our study, we adapted the TBS, which has previously been used to assess the effect of training of robotic cardiac skills in wet lab settings.^16^ TBS assessment tool coincided with the mGEARS score in distinguishing between different levels of experience, demonstrating a significant difference among the novices and experienced surgeons at the baseline for the atrial closure and mitral stitches tasks, but alike mGEARS did not reach the defined level of significance for the ITA harvesting task. The reliability of the score based on the test/retest reliability was highest for the atrial closure and lowest for the ITA dissection. These findings encourage the use of TBS as a reliable assessment tool to measure competency in the 2 former tasks.

The established pass/fail scores for the atrial closure and mitral stitches tasks discriminated reliably between the novices and experienced surgeons, allowing only a few passing novices and failing experts for both tasks, even after adjusting for a small sample size.^18^ Combining the 2 levels from both scores increased the ability to discriminate between different levels of experience even further, nearly eliminating failing experts and passing novices for the latter tasks. The proposed cut-off levels for mGEARS and TBS can be incorporated into proficiency-based curricula for robotic cardiac surgery wet-lab simulation training and can be used for credentialing in preclinical settings. The pass/fail score for the ITA harvesting task yielded a high number of theoretical false positives on TBS and a high number of theoretical false negatives on the mGEARS score. Alongside the insufficient validity evidence supporting the assessment of the ITA exercise, the determined pass/fail score appears to be an unreliable discriminator between competent and non-competent operators and warrants further investigation before it can be implemented for assessment purposes.

The porcine wet lab set-up described in the details in the study along with the investigated tasks demonstrated on the Video 1 can relatively easily be reproduced by other institutions making our work scalable and our pass/fail cut-off levels for TBS and mGEARS scores usable for other institutions. The results of the study should be considered as a starting point for standardization of robotic cardiac surgery skill testing.

### Study Limitations

Even though, at the time of publication, this study includes the largest number of participants in a robotic cardiac wet lab simulation, the small sample size remains one of the major limitations, especially regarding the IMA harvesting task. Moreover, we did not train the raters in mGEARS assessment before the study began because we wished to explore the validity of mGEARS in the hands of untrained clinician supervisors. This could in part explain the high systemic variation among the raters. Finally, the time constraints of surgical participants included in the study from different international sites and departments forced us to choose only 5 mitral stitches. However, as both rings and bands are currently used in robotic mitral surgery depending on the surgeon's preference, the number of sutures either within the whole mitral annulus or only within the posterior annulus can be discussed. The proposed 5 sutures could be adopted for the sake of standard setting for credentialing.

## Conclusions

This study provides sufficient evidence for using TBS and mGEARS scores in evaluating robotic-assisted cardiac surgery skills in wet lab settings for intracardiac tasks. Combining both assessment tools enhances the evaluation of proficiency in robotic cardiac surgery, paving the way for standardized, evidence-based preclinical training and credentialing. We strongly recommend the incorporation of the standardized wet lab protocols used in this study into robotic cardiac surgery training programs to allow for improved efficiency of skill acquisition and to ensure better and safer surgical performances.

## Conflict of Interest Statement

The authors reported no conflicts of interest.

The *Journal* policy requires editors and reviewers to disclose conflicts of interest and to decline handling or reviewing manuscripts for which they may have a conflict of interest. The editors and reviewers of this article have no conflicts of interest.

## Supplementary Data

Video 1 Simulation-based assessment of robotic cardiac surgery skills. Video available at: https://www.jtcvs.org/article/S2666-2736(23)00347-9/fulltext.
