# Supplementary figures and images for: Simulation-based assessment of robotic cardiac surgery skills: An international multicenter, cross-specialty trial
Source: JTCVS Open. 2023 Nov 2;16:619–27. doi: 10.1016/j.xjon.2023.10.029 (PMC10775167; doi:10.1016/j.xjon.2023.10.029)

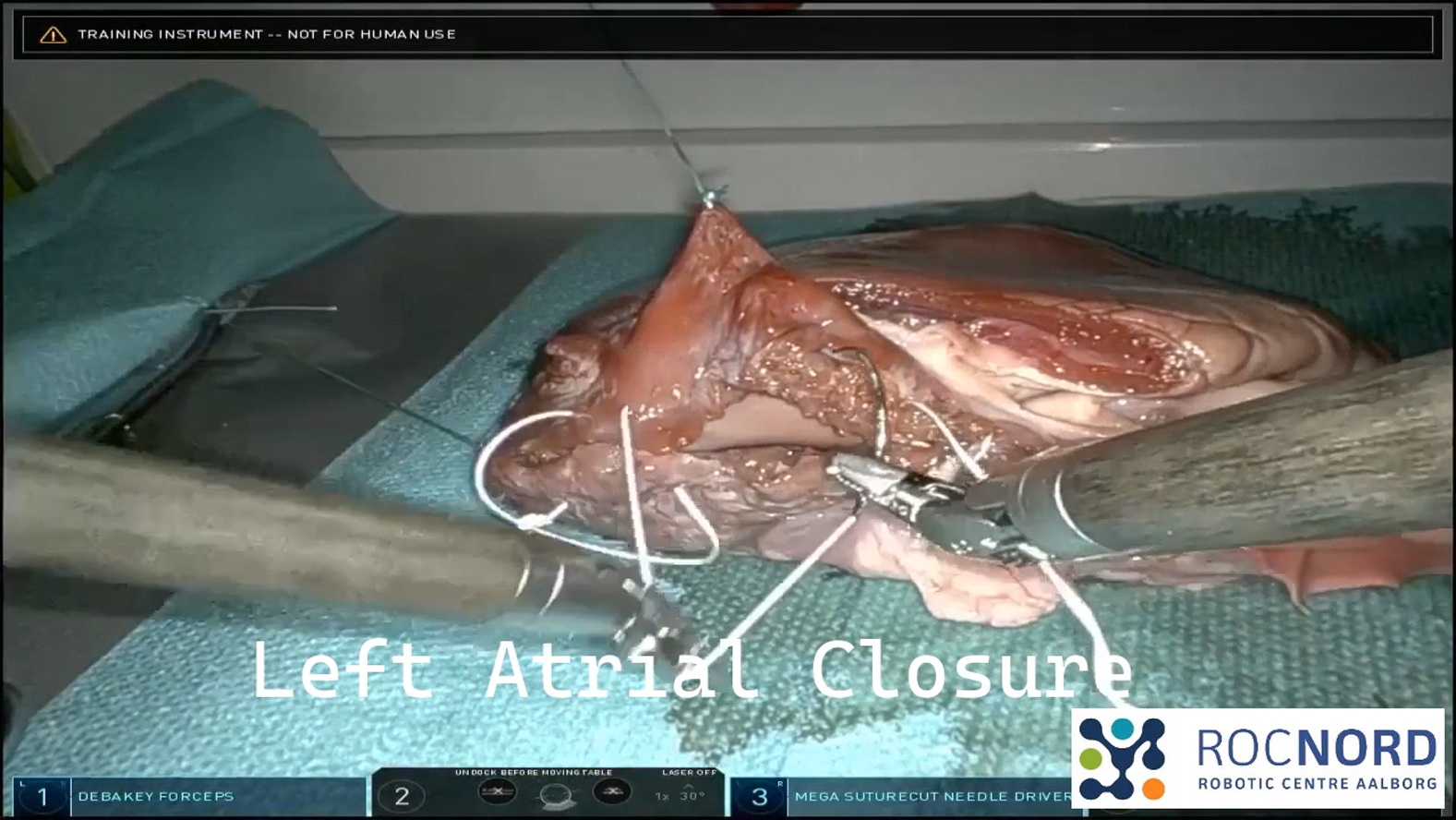

Supplement: Video 1 — Simulation-based assessment of robotic cardiac surgery skills. Video available at: https://www.jtcvs.org/article/S2666-2736(23)00347-9/fulltext. [file fx2.jpg]
